# Correlation of Pre-Hypertension with Carotid Artery Damage in Middle-Aged and Older Adults

**DOI:** 10.3390/ijerph17207686

**Published:** 2020-10-21

**Authors:** Jinkee Park, Yongseong Na, Yunjung Jang, Song-Young Park, Hyuntae Park

**Affiliations:** 1 Department of Sport Rehabilitation, Dong-Ju College, Busan 49318, Korea; park7166@gmail.com; 2 Department of Health Science, Dong-A University, Busan 49315, Korea; Solpidem@daum.net (Y.N.); yunjung2005@hanmail.net (Y.J.); 3 School of Health and Kinesiology, University of Nebraska Omaha, Omaha, NE 68182, USA; song-youngpark@unomaha.edu; 4 Institute of Convergence Bio-Health, Dong-A University, Busan 49315, Korea

**Keywords:** pre-hypertension, carotid artery structure, flow velocity, compliance and stiffness, carotid artery damage, middle-aged and older adults

## Abstract

The intima–media thickness (IMT), luminal diameters (LDs), flow velocities (FVs), compliance, and β-stiffness of the carotid artery (CA) are considered as independent risk factors for cardiovascular diseases (CVDs). Pre-hypertension (PHT) is also an independent CVD risk factor. This study investigated the association between CA damage (CAD) and PHT. A total of 544 adults participated; their blood pressures (BPs) and CA characteristics were measured using a mercury-free sphygmomanometer and ultrasound. Analysis of covariance (ANCOVA) was performed to assess the differences in the CA characteristics according to the BPs, multinomial logistic regression to evaluate the risk of CAD associated with PHT. In ANCOVA, the CA characteristics of PHT were significantly different from normotensive. The odds ratios (ORs) of IMTmax, LDmax, LDmin, peak-systolic FV (PFV), end-diastolic FV (EFV), PFV/LDmin, EFV/LDmax, compliance, and β-stiffness of PHT were 4.20, 2.70, 3.52, 2.41, 3.06, 3.55, 3.29, 2.02, and 1.84 times higher than those of the normotensive, respectively, in Model 2. In Model 3 adjusted for age, the ORs of LDmax, LDmin, EFV, PFV/LDmin, and EFV/LDmax of PHT were 2.10, 2.55, 1.96, 2.20, and 2.04 times higher than those of the normotensive, respectively. Therefore, the present study revealed that CAD is closely correlated with pre-hypertensive status in adults.

## 1. Introduction

Carotid artery intima–media thickness (IMT) has been known to associate with being an early marker of atherosclerosis, as well as closely associated with the early incidence of cardiovascular disease (CVD) and ischemic stroke in a wide age range [1,2,3,4]. Moreover, carotid artery IMT can strongly predict the occurrence of CVD and coronary artery disease [4,5,6]. Similarly, the carotid artery luminal diameter (LD) is closely associated with the early incidence of CVD and stroke [7,8]. Furthermore, previous cross-sectional studies have reported that low peak-systolic flow velocity (PFV) and end-diastolic flow velocity (EFV) are risk factors for the development of atherosclerosis and stroke [7,9], and another recent cross-sectional study reported that a low carotid PFV/maximum LD (LD_max_) ratio is related to CVD events in hypertension independent of IMT [10].

The effect of hemodynamics on the carotid artery has been confirmed through a number of in vitro studies [11], and numerical fluid biomechanics is known as an effective tool for understanding vascular diseases including carotid artery atherosclerosis [12]. Ultrasound evaluation of the carotid artery is a noninvasive way to identify IMT along with other vascular characteristics such as luminal diameter (LD), flow velocity, and arterial function [1,6,9]. In addition, the possibility of a diagnosis of atherosclerosis of the carotid artery by ultrasound has been simulated through several studies [13].

Hypertension, a chronic disease with a high prevalence not only in Asia but also worldwide [14], is closely associated with metabolic risk factors such as obesity, insulin resistance, and abnormal lipid profiles in adults [15,16]. In addition, pre-hypertension has a high prevalence in Asia [17,18]. Pre-hypertension is reported to be associated with damage of subclinical cardiac dysfunction and CVD target organs [19,20].

Meanwhile, in studies that investigate the relationship between blood pressure and carotid artery, hypertension is reported to be associated with damage to carotid artery characteristics such as IMT and LD and large arterial stiffness [21,22,23,24,25].

Although the relationship between structural alterations of carotid artery and the development of CVD has drawn interests, only two studies examined the association between the carotid artery and pre-hypertension [15,23]. Furthermore, the previous two studies showed different results in terms of the relationship between prehypertension status and IMT. This discrepancy may be due to small sample size and different subjects characteristics (age, sex, drug use, etc). In addition, correlations among LD, flow parameters, and function in the carotid artery with CAD and stroke have been reported [7,8,9,10], but there are no reports on the relationship between prehypertension and the carotid artery parameters. Therefore, a study which examines association between pre-hypertension and other structural characteristics in the carotid artery such as LD, flow velocities, compliance, and stiffness is needed. We investigated the association between pre-hypertension and the overall carotid artery structural characteristics.

## 2. Experimental Section

### 2.1. Study Participants

This cross-sectional study was performed between July 2015 and June 2016 in South Korea. A total of 609 adults (age range, 19–85 years) applied to participate in the study. The study details were explained in community health centers, sports centers, and at an elderly welfare center. The primary exclusion criterion was <20 years; 15 participants were excluded. Additionally, those who did not complete all the tests required in this study were also excluded and considered as secondary exclusion criterion, comprising 50 participants (blood pressure, *n* = 12; body composition, *n* = 14; carotid artery ultrasound, *n* = 14; and missing questionnaire, *n* = 10). Finally, we analyzed the data of 544 adults (mean age, 55.2 ± 18.2 years, Table 1) and classified the patients as normotensive (<120/80 mmHg), pre-hypertensive (120–139/80–89 mmHg), and hypertensive (≥140/90 mmHg or use of antihypertensive drugs) [21,26]. In our study, the blood pressure classification was based on Korean hypertension guidelines [26]. This criterion is consistent with the Joint National Committee (JNC) 7 [27]. The blood pressure classification criteria in our study are different from the new blood pressure classification criteria reported by the American College of Cardiology (ACC)/American Heart Association (AHA) [28]. Recently, Ihm et al. reported that applying the new blood pressure standard to Asians may not be appropriate [27].

CVD (angina, myocardial infarction), stroke, diabetes, hyperlipidemia, lung disease, and musculoskeletal disorders were defined as the indications for current use of medications. Information on clinical outcomes, alcohol drinking, and smoking was obtained through direct questions. Physical activity was measured using the Korean short version of the International Physical Activity Questionnaire (IPAQ, available at https://sites.google.com/site/theipaq/). We obtained informed consent from all participants before their enrollment into the study. The study was conducted in accordance with the Declaration of Helsinki, and it was approved by the Institutional Review Board of Dong-A University (2-104709-AB-01-201505-HR-014-04) and Korea National Institute for Bioethics Policy (P01-201801-003).

### 2.2. Body Composition and Blood Pressure Assessment

After measuring the height and body mass using a body composition analyzer (Inbody 370; Biospace, Seoul, Korea), we measured the body fat mass (BFM %) and lean body mass (LBM) using bioelectrical impedance analysis. The approximate waist circumference was measured (in cm) at the level of the umbilicus using a flexible plastic tape with the participant in the standing position. Hip circumference was measured at the widest circumference over the buttocks followed by calculation of the waist-to-hip ratio (WHR) and body mass index (BMI). Two days before body composition measurement, the participants were instructed to consume light meals and avoid physically exerting activities. Systolic blood pressure (SBP) and diastolic blood pressure (DBP) were measured using a mercury-free sphygmomanometer (CK-E301, Spirit Medical Co., Taiwan) after a 10-min rest, and the precision was ensured based on the reproducibility of blood pressure measurements through a test–retest of 38 participants. The Pearson correlation coefficients between the test–retest results were 0.85 and 0.81 for the SBP and DBP, respectively.

### 2.3. Carotid Artery Characteristics Assessment

Carotid artery characteristics were assessed using B-mode ultrasound with a 10-MHz probe (LOGIQ 3; GE Healthcare, Wauwatosa, WI, USA). While evaluating the left carotid artery using ultrasound, the IMT and LD were measured from the far wall of the distal common carotid artery, 1–3 cm proximal to the carotid bifurcation. We defined IMT as the distance from the lumen–intima interface to the intima–adventitia interface; three points were measured and the thickest point was IMT_max_, the thinnest point was IMT_min_, and the average was IMT_mean_ [10,29]. Similarly, we defined LD as the distances between the near-and-far wall and the intima–media interfaces, the LD_max_, and the minimum LD (LD_min_) were obtained by continuously tracing five cycles and considering the average value [10,29]. For spectral Doppler analysis, the PFV and EFV were measured using continuous-wave Doppler examination of the common carotid artery, 1–2 cm proximal to the bifurcation [9,29]. The carotid artery compliance (CAC) and β-stiffness index of the carotid artery were calculated using previously described equations [9,30]. Finally, the IMT_max_/LD_max_, PFV/LD_min_, and EFV/LD_max_ ratios were calculated and the reproducibility was assessed through test–retest on 38 participants. The Pearson correlation coefficients between the test–retest results were 0.85, 0.80, and 0.76 for the IMT, LD, and PFV in the carotid artery, respectively.

### 2.4. Statistical Analysis

SPSS ver. 17.0 (SPSS Inc., Chicago, IL, USA) was used for the statistical analysis, and the data were presented as averages, standard deviations (SD), and frequencies. Analysis of variance (ANOVA) was used to evaluate differences in the baseline characteristics and those of the carotid artery among different blood pressure groups (normotensive, pre-hypertensive, and hypertensive) and age groups (19–39 years, 40–59.9 years, and ≥60 years) [21]. If a significant effect was found, a post-hoc test was performed according to Scheffe’s method. We performed an analysis of covariance (ANCOVA) to evaluate the differences in carotid artery parameters among different blood pressure groups, after adjusting for risk factors including CVD, stroke, lung disease, musculoskeletal disorders, diabetes, hyperlipidemia, current alcohol drinking and smoking habits, sex, physical activity, BMI, BFM%, LBM, WHR, and age. 

We performed multinomial logistic regression to evaluate the risk of carotid artery damage considering the pre-hypertensive and hypertensive groups after multivariate adjustments. In our study, indices of carotid artery damage were IMT, LDs (max and min), flow velocities (PFV, EFV, and PFV/LDmin and EFV/LDmax ratios), compliance, and stiffness. The IMT cut-off value was set at over 1.00 mm for assessing the CVD risk, as evaluated by previous studies [31,32,33,34]. The cut-off values for carotid artery LDmax and LDmin, flow velocities (PFV, EFV, and PFV/LDmin and EFV/LDmax ratios), compliance, and stiffness are unclear. Therefore, after calculating quartiles for each carotid artery variable, we set the cut-off value as 75% for LDs and stiffness and 25% for flow velocities, PFV, EFV, and PFV/LDmin and EFV/LDmax ratios, and compliance. The statistical significance level was set at *p* < 0.05.

## 3. Results

In this study, the number of normotensive, pre-hypertensive, and hypertensive patients were 274 (50.4%), 150 (27.6%), and 120 (22.1%), respectively. Table 1 shows the differences in baseline characteristics of all participant groups classified by blood pressure. Age, height, BMI, BFM%, WHR, LBM, SBP, and DBP were significantly different among groups. The age, height, BMI, BFM%, WHR, SBP, DBP, and physical activity of the pre-hypertensive and hypertensive groups were significantly different from those of the normotensive group. The hypertensive group and the normotensive group showed significant differences in LBM. The age, BFM%, WHR, SBP, DBP, and physical activity of the hypertensive group were significantly different from those of the pre-hypertensive group. However, body mass was not different between groups, and the LBM of the pre-hypertensive and the normotensive groups had no significant differences.

Table 2 shows the coefficient of Pearson’s correlations between body composition and carotid arterial characteristics with blood pressure. SBP had significantly positive correlations with age, body mass, BMI, BFM%, and WHR, and it was negatively correlated with physical activity. DBP showed significant positive correlations with age, body mass, BMI, BFM%, and WHR. Moreover, SBP showed significant positive correlations with carotid IMTs (max, mean, and min), LDs (max and min), IMT/LD_max_, and the ꞵ-stiffness index. SBP was negatively correlated with PFV, EFV, PFV/LD_min_, EFV/LD_max_ ratios, and compliance. Furthermore, DBP showed significant positive correlations with IMTs (max, mean, and min) and LD_min_; it was significantly negatively correlated with PFV, PFV/LD_min_ ratio, and the compliance.

**Figure 1:**
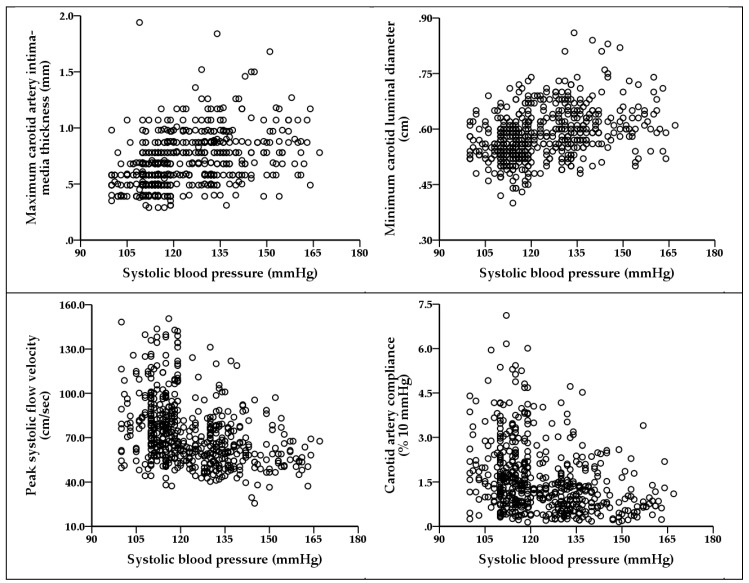
This scatterplot matrix figure illustrates the association (Pearson correlation coefficient, *p* value) between systolic blood pressure (SBP) and carotid artery intima–media thickness (r = 0.400, *p* < 0.001); SBP and minimum carotid artery luminal diameter (r = 0.308, *p* < 0.001); SBP and peak systolic flow velocity (r = −0.361, *p* < 0.001); and SBP and carotid artery compliance (r = −0.307, *p* < 0.01).

Figure 1 shows the scatterplots for association between SBP and carotid IMTmax, LDmin, PFV, and carotid artery compliance.

Table 3 shows the different carotid artery characteristics among the different blood pressure groups after multivariate adjustment. The ANOVA results show that the IMTs, LDs, IMT_max_/LD_max_, PFV, EFV, PFV/LD_min_ and EFV/LD_max_ ratios, compliance, and β-stiffness index were significantly different among groups (*p* < 0.001). The overall carotid artery characteristics of the pre-hypertensive and hypertensive groups were significantly different from those of the normotensive group. The IMTs, LDs, EFV, and EFV/LDmax ratio of the hypertensive group were significantly different from those of the pre-hypertensive group. From ANCOVA, the IMT_max_, IMT_mean_, IMT_min_, LD_max_, LD_min_, PFV, EFV, PFV/LD_min_, and EFV/LD_max_ were significantly different among groups after multivariate adjustment (*p* < 0.001).

Table 4 shows the carotid artery characteristics among groups according to the category of blood pressure and age. Within all age groups, carotid artery structure and flow velocity parameters were significantly different among groups according to the blood pressure category.

Table 5 presents the results of the multinomial logistic regression analysis, showing that pre-hypertensive and hypertensive were associated to the carotid artery damage after multivariate adjustment. After excluding age from the multivariable adjustment, pre-hypertensive and hypertensive groups were at a significantly higher risk of damage in all carotid artery parameters compared to the normotensive groups. Furthermore, when age was added to the covariates, the hypertensive group had a significantly higher risk of damage in all carotid artery parameters compared to the normotensive group; the pre-hypertensive group had a significantly higher risk of carotid artery damage in terms of IMT, LDs (max and min), EFV, and ratios of PFV/LDmin and EFV/LDmax than the normotensive group.

## 4. Discussion

It is well established that hypertension is a medical condition that can possibly increase the risks of a multitude of CVD. This study examined the association of carotid artery characteristics with pre-hypertension in adults. The new findings of the present study were that being in the pre-hypertensive category, as well as hypertensive category, can negatively change in structures (IMTs, LDs, compliance, and stiffness) and flow velocities (PFV, EFV, and ratios of PFV/LD_min_ and EFV/LD_max_ of the carotid artery). These results likely have clinical significance and could also potentially contribute to early diagnosis and prognosis of pre-hypertension. Although the methods used in the present study are subclinical measures, these measures could potentially provide additional non-invasive clinical markers to monitor the disease. Being able to track changes in structures and flow velocities could potentially allow health professionals to track the progression of the disease more accurately and prevent potential development of CVD.

The association between pre-hypertension with risk factors for CVD and metabolic disease has been confirmed through various previous studies [16,18,23]. Our study investigated the association between pre-hypertension and carotid artery characteristics. Several studies examining the association between blood pressure and carotid artery characteristics demonstrated that hypertension is associated with carotid artery IMT [15,22,23,35,36]. Additional studies have also shown that hypertension was associated with diameter, flow velocity, and function in the carotid artery [24,25,37].

Meanwhile, the associations between pre-hypertension and diameter, flow velocity, and function in the carotid artery are still unclear. Previous studies on the relationship between pre-hypertension and large artery structure (especially the diameter) showed some differences [37,38]. Furthermore, carotid artery LD, flow velocity, and function are reported as independent risk factors for CVD and stroke [7,9,39,40]. Our study showed that being in the pre-hypertensive category was associated with the carotid artery structure, flow velocity, and function. This association was also confirmed in the covariance analysis which adjusted for covariates including age. Our results suggest that not only hypertension but also pre-hypertension is associated with negative changes in carotid artery characteristics. If we were to create cut off values that could indicate early carotid artery abnormalities, they would be found in Table 3 within the pre-hypertensive column. However, in interpreting the general association between pre-hypertensive and carotid artery characteristics, further studies are warranted, and the limitations of our study should be considered. There is an age difference between normotensive and pre-hypertensive in our study. Age is reported to be related to blood pressure and carotid artery characteristics [41,42,43,44]. Despite the fact that we controlled for age in our study, perhaps age has influenced our results. Several similar previous studies reported that there was a significant correlation between blood pressure and carotid artery IMT, despite a large age difference, and an association between flow velocity and carotid artery IMT [21,41,45]. In addition, in our study, we classified three groups according to age and then examined differences in carotid artery characteristics according to blood pressure within each group. Differences in carotid artery characteristics according to blood pressure were found in all age groups. Therefore, we believe that pre-hypertension is associated with carotid artery characteristics. However, additional research is needed to establish a clear association.

The development of clinical imaging and numerical capabilities allows researchers and clinicians to access vascular biomechanics from various aspects [12]. In addition, many researchers are simulating human arteries (e.g., carotid artery bifurcation) to understand the biomechanical behaviors of carotid arteries in CVD [11,13]. For this reason, simulation of carotid artery arteriosclerosis and numerical fluid biomechanics are reported as effective tools for evaluating atherosclerotic diseases such as CVD [11,13]. The IMT, LD, flow velocity, and stiffness of carotid arteries are reported as independent risk factors for CVD in a wide range of subjects [2,7,8]. Moreover, high blood pressure is associated with negative changes in carotid artery characteristics in adults [24,35,46]. Based on previous studies, we performed logistic regression analysis to assess the risk of carotid damage using carotid artery characteristics and blood pressure [2,7,8,24,35,46]. When the carotid artery characteristics were categorized into two groups according to cut-off, the adjusted odds ratio (OR) and 95% confidence interval (CI) calculated by logistic regression analysis showed significant relationships between the estimated vulnerability to carotid artery damage and the level of carotid artery characteristics and blood pressure. Therefore, we believe that pre-hypertension is associated with carotid artery damage.

A carotid artery IMT ≥ 1.00 mm was used in previous studies as a cut-off for the risk of CVD [31,32,33]. We used this value as the cut-off value for carotid artery damage by IMT. Meanwhile, the cut-off points for carotid artery LD, flow velocities, and ratios of PFV/LDmin and EFV/LDmax remain unclear. It was judged that the values suggested in the study by Bellinazzi et al. were not suitable for the application in our study [10]. We set the upper 75% and lower 25% as the cut-off values for carotid artery damage after calculating quartiles for other carotid artery parameters excluding IMT.

In Model 3 of the logistic analysis, LDmax, LDmin, PFV/LDmin, and EFV/LDmax of pre-hypertensive had a significantly higher risk of carotid artery damage compared to normotensive. Aging is known as one of several factors that affect negative changes in the carotid artery [47,48]. However, our results suggest that carotid artery LD and flow velocity are associated with pre-hypertensive, even considering the effects of age, and pre-hypertensive is associated with a high risk of carotid artery damage. In our study, the pre-hypertensive group was older and significantly different from the normal blood pressure one. Although age was controlled for in our study, it still appears to be a limitation of the study. We need to consider the limitations of our study when understanding the association between pre-hypertension and carotid artery characteristics.

There are a few limitations to our study. In our study, CVD-related biochemical markers such as serum lipids, glucose, and C-reactive protein were not examined. Several studies demonstrated an association between carotid artery characteristics and CVD-related biochemical markers [9,45,49]. In our study, there was a significant age difference between pre-hypertensive and normotensive individuals. This difference can be a factor in our results. In our study, there was a significant age difference between pre-hypertension and normotensive groups. Aging is reported to be related to carotid artery characteristics [42,43]. Meanwhile, in our study, differences in carotid artery characteristics according to blood pressure were found in all age groups (Table 4). Nevertheless, the characteristics of the subjects are considered to be the limitations of our study. Moreover, we used the IPAQ to examine information on individual patient history related to biomarkers and used them as control variables in our analysis.

## 5. Conclusions

Before this present study, there are no clear relationships between pre-hypertension and characteristics such as lumen diameter, flow velocities, compliance, and stiffness of the carotid artery. A non-invasive technique was used to examine these characteristics and the relationship between carotid artery and pre-hypertension.

The results of this study reveal for the first time that pre-hypertension is closely correlated with structural changes of the carotid artery. Additionally, intima–media thickness, lumen diameter, flow velocities, compliance, and stiffness of the carotid artery found by ultrasound imaging technique may be useful non-invasive diagnostic measures to prevent potential development of atherosclerosis in pre-hypertensive adults.

## Figures and Tables

**Table 1:** Differences in general characteristic among groups according to the blood pressure category.

Variable	Total (*n* = 544)	Normotensive (*n* = 274)	Pre-Hypertensive (*n* = 150)	Hypertensive (*n* = 120)	*p*-Value
Sex, male/female	83/461	43/231	17/133	23/97	
Age, years	55.2 ± 18.2	46.3 ± 16.3	62.0 ± 16.4 #	67.0 ± 13.7 # ƒ	<0.001
Height, cm	158.7 ± 7.2	159.9 ± 7.7	157.8 ± 6.8 #	157.1 ± 6.5 #	<0.001
Body mass, kg	60.3 ± 9.2	59.5 ± 9.1	61.3 ± 9.4	60.8 ± 8.8	
Body mass index, kg/m^2^	23.9 ± 3.0	23.2 ± 2.9	24.6 ± 3.1 #	24.6 ± 2.9 #	<0.001
Body fat mass, %	32.0 ± 8.2	29.7 ± 7.6	33.8 ± 8.1 #	35.3 ± 7.7 # ƒ	<0.001
Lean body mass, kg	40.8 ± 7.8	41.7 ± 8.2	40.4 ± 7.6	39.0 ± 6.7 #	<0.001
Waist to hip ratio	0.89 ± 0.1	0.86 ± 0.1	0.90 ± 0.1 #	0.92 ± 0.1 # ƒ	<0.001
Systolic blood pressure, mmHg	123.8 ± 14.5	112.5 ± 5.0	129.9 ± 6.3 #	142.1 ± 13.1 # ƒ	<0.001
Diastolic blood pressure, mmHg	76.3 ± 8.1	72.4 ± 5.5	79.0 ± 7.2 #	81.8 ± 9.7 # ƒ	<0.001
Physical activity, Mets min/week	1923.0 ± 2195.3	2935.4 ± 2497.5	1667.4 ± 1849.7 #	1163.9 ± 1490.1 # ƒ	<0.001
Cardiovascular disease, *n*	10	6	2	2	
Stroke, *n*	6	3	1	2	
Lung disease, *n*	7	4	1	1	
Musculoskeletal disorders, *n*	78	10	32	36	<0.001
Hypertension, *n*	120			120	<0.001
Diabetes, *n*	33	11	29	6	<0.001
Hyperlipidemia, *n*	52	28	12	12	<0.042
* Alcohol drinking, *n*	403	217	107	79	<0.014
* Smoking, *n*	81	33	18	30	<0.002

Values are expressed as mean ± SD; Category for blood pressure: normotensive, <120/80 mmHg; pre-hypertensive, 120–139 or 80–89 mmHg; hypertensive, ≥140 or ≥90 mmHg and using antihypertensive drugs. # indicates the significant difference compared with Normotensive, *p* < 0.05; ƒ indicates the significant difference compared with pre-hypertensive, *p* < 0.05; * indicates that the current smoking and drinking habits were evaluated using the questionnaire.

**Table 2:** Coefficient of Pearson’s correlations between body composition and carotid arterial characteristics with blood pressure.

Variable	Systolic Blood Pressure, mmHg	Diastolic Blood Pressure, mmHg
Age, years	0.464 ***	0.093 *
Body mass index, kg/m^2^	0.257 ***	0.203 ***
Body fat mass percent, %	0.276 ***	0.149 ***
Lean body mass, kg	−0.069	0.007
Waist to hip ratio	0.372 ***	0.181 ***
Physical activity, Mets min/week	0.200 ***	0.026
Intima-media thickness max, mm	0.400 ***	0.115 **
Intima-media thickness min, mm	0.423 ***	0.136 **
Intima-media thickness mean, mm	0.421 ***	0.127 **
Luminal diameter max, cm	0.308 ***	0.080
Luminal diameter min, cm	0.367 ***	0.138 **
IMT/luminal diameter max	0.330 ***	0.069
Peak-systolic flow velocity, cm/sec	−0.361 ***	−0.184 ***
End-diastolic flow velocity, cm/sec	−0.366 ***	−0.070
PFV/luminal diameter max, s^−1^	−0.400 ***	−0.205 ***
EFV/luminal diameter min, s^−1^	−0.393 ***	−0.091 *
Carotid artery compliance, % 10 mmHg	−0.307 **	−0.166 ***
β-stiffness index, AU	0.231 ***	0.023

PFV, peak-systolic flow velocity; EFV, end-diastolic flow velocity; * *p* < 0.05, ** *p* < 0.1, *** *p* < 0.001.

**Table 3:** Differences in carotid arterial characteristics among groups according to the blood pressure category after multivariate adjustment.

Variable	ANOVA				ANCOVA					
	Normotensive (*n* = 274)	Pre-Hypertensive (*n*= 150)	Hypertensive (*n* = 120)	Crude	Model 1		Model 2		Model 3	
				*p*-Value	F-Value	*p*-Value	F-Value	*p*-Value	F-Value	*p*-Value
IMT_max_, mm	0.59 ± 0.2	0.77 ± 0.2 #	0.85 ± 0.2 # ƒ	<0.001	40.689	<0.001	25.505	<0.001	8.780	<0.001
IMT_mean_, mm	0.55 ± 0.2	0.71 ± 0.2 #	0.77 ± 0.2 # ƒ	<0.001	44.797	<0.001	28.356	<0.001	10.153	<0.001
IMT_min_, mm	0.51 ± 0.1	0.65 ± 0.2 #	0.70 ± 0.2 # ƒ	<0.001	43.694	<0.001	27.439	<0.001	10.240	<0.001
LD_max_, cm	0.60 ± 0.1	0.64 ± 0.1 #	0.67 ± 0.1 # ƒ	<0.001	22.772	<0.001	16.159	<0.001	11.829	<0.001
LD_min_, cm	0.56 ± 0.1	0.60 ± 0.1 #	0.63 ± 0.1 # ƒ	<0.001	35.800	<0.001	24.619	<0.001	15.531	<0.001
IMT_max_/LD_max_	0.93 ± 0.3	1.19 ± 0.4 #	1.25 ± 0.4 #	<0.001	23.055	0.097	13.687	<0.001	2.557	0.078
PFV, cm/s	83.1 ± 23.5	66.1 ± 17.1 #	61.5 ± 14.5 #	<0.001	39.778	0.001	25.341	<0.001	7.760	<0.001
EFV, cm/s	25.0 ± 4.7	21.3 ± 5.1 #	19.7 ± 5.9 # ƒ	<0.001	25.959	<0.001	16.944	<0.001	9.284	<0.001
PFV/LD_min_, s^−1^	151.8 ± 49.6	112.8 ± 35.5 #	100.5 ± 29.5 #	<0.001	48.966	<0 001	31.493	<0.001	12.261	<0.001
EFV/LD_max_, s^−1^	41.9 ± 9.4	34.1 ± 9.9 #	30.4 ± 10.9 # ƒ	<0.001	34.334	<0.001	23.197	<0.001	14.141	<0.001
CAC, % 10 mmHg	1.95 ± 1.3	1.31 ± 1.0 #	1.15 ± 0.8 #	<0.001	18.218	<0.001	10.614	<0.001	2.731	0.066
β-stiffness index, AU	8.9 ± 8.2	12.5 ± 10.0 #	14.1 ± 12.1 #	<0.001	8.487	<0.001	5.513	0.004	1.815	0.164

Values are mean ± SD; IMT, intima–media thickness; LD_max_, maximum luminal diameter; LD_min_, minimum luminal diameter; PFV, peak-systolic flow velocity; EFV, end-diastolic flow velocity; CAC, carotid artery compliance. Category for blood pressure: normotensive, <120/80 mmHg; pre-hypertensive, 120–139 or 80–89 mmHg; hypertensive, ≥140 or ≥90 mmHg. Crude, not adjusted. Three models were created for multivariate adjustments in the ANOVA and ANCOVA: Model 1, adjusted for cardiovascular disease, stroke, lung disease, musculoskeletal disorders, diabetes, hyperlipidemia, current alcohol drinking and smoking, and sex; Model 2, adjusted for Model 1, pulse, body mass index, body fat%, lean body mass, waist to hip ratio, and physical activity. Model 3, adjusted for Models 1 and 2, pulse, and age. # *p* < 0.05, difference vs. normotensive; ƒ *p* < 0.05, difference vs. pre-hypertensive.

**Table 4:** Differences in carotid arterial characteristics among groups according to the category of blood pressure and age.

Variable	19–39 Years				40–59 Years				≥60 Years			
	Normotensive (*n* = 78)	Pre-Hypertensive (*n* = 12)	Hypertensive (*n* = 6)	*p*-Value	Normotensive (*n* = 144)	Pre-Hypertensive (*n*= 49)	Hypertensive (*n* = 35)	*p*-Value	Normotensive (*n* = 52)	Pre-Hypertensive (*n* = 89)	Hypertensive (*n* = 79)	*p*-Value
IMT_max_, mm	0.45 ± 0.1	0.54 ± 0.2	0.55 ± 0.0	0.001	0.60 ± 0.1	0.64 ± 0.1	0.68 ± 0.1	0.016	0.78 ± 0.2	0.88 ± 0.2	0.95 ± 0.2	<0.001
IMT_mean_, mm	0.42 ± 0.1	0.52 ± 0.1	0.51 ± 0.0	<0.001	0.56 ± 0.1	0.60 ± 0.1	0.63 ± 0.1	0.020	0.70 ± 0.1	0.80 ± 0.2	0.86 ± 0.2	<0.001
IMT_min_, mm	0.39 ± 0.1	0.49 ± 0.1	0.48 ± 0.1	<0.001	0.52 ± 0.1	0.55 ± 0.1	0.58 ± 0.1	0.048	0.63 ± 0.1	0.72 ± 0.1	0.77 ± 0.2	<0.001
LD_max_, cm	0.60 ± 0.1	0.62 ± 0.0	0.64 ± 0.0	0.168	0.60 ± 0.1	0.62 ± 0.1	0.63 ± 0.1	0.008	0.62 ± 0.1	0.65 ± 0.1	0.68 ± 0.1	<0.001
LD_min_, cm	0.53 ± 0.1	0.57 ± 0.0	0.59 ± 0.0	0.004	0.56 ± 0.1	0.58 ± 0.1	0.60 ± 0.1	<0.001	0.58 ± 0.07	0.61 ± 0.07	0.64 ± 0.08	<0.001
IMT_max_/LD_max_	0.72 ± 0.1	0.76 ± 0.2	0.82 ± 0.2	0.026	0.96 ± 0.3	0.98 ± 0.3	1.01 ± 0.3	0.528	1.20 ± 0.30	1.40 ± 0.33	1.40 ± 0.41	0.008
PFV, cm/s	104.2 ± 23.1	98.1 ± 18.6	81.0 ± 17.2	0.044	77.4 ± 18.2	69.1 ± 17.3	69.6 ± 14.1	0.004	67.1 ± 13.9	60.1 ± 10.4	56.5 ± 11.5	<0.001
EFV, cm/s	25.3 ± 4.1	25.4 ± 2.2	24.0 ± 5.4	0.733	25.7 ± 4.4	23.0 ± 4.9	24.1 ± 3.7	0.001	22.4 ± 4.9	19.8 ± 5.0	17.4 ± 5.5	<0.001
PFV/LD_min_, s^−1^	196.6 ± 47.4	174.1 ± 32.8	138.5 ± 33.1	0.006	139.8 ± 38.5	121.4 ± 37.8	117.8 ± 28.0	<0.001	118.2 ± 31.7	99.8 ± 22.2	89.9 ± 23.7	<0.001
EFV/LD_max_, s^−1^	42.5 ± 8.3	40.8 ± 4.1	37.7 ± 9.1	0.320	43.2 ± 8.9	37.5 ± 9.1	38.6 ± 7.3	<0.001	36.9 ± 10.2	31.3 ± 10.0	26.1 ± 9.9	<0.001
CAC, % 10 mmHg	2.9 ± 1.5	2.2 ± 1.3	1.8 ± 0.9	0.100	1.7 ± 1.1	1.5 ± 1.0	1.3 ± 0.9	0.087	1.4 ± 0.8	1.1 ± 0.7	1.1 ± 0.7	0.047
β-stiffness index, AU	5.5 ± 5.2	7.0 ± 6.9	7.2 ± 4.1	0.557	9.4 ± 7.3	9.5 ± 5.6	13.1 ± 9.5	0.023	12.7 ± 11.8	14.9 ± 11.4	15.1 ± 13.2	0.509

Values are mean ± SD; IMT, intima–media thickness; LD_max_, maximum luminal diameter; LD_min_, minimum luminal diameter; PFV, peak-systolic flow velocity; EFV, end-diastolic flow velocity; CAC, carotid artery compliance. Category for blood pressure (systolic and diastolic pressure): normotensive, <120/80 mmHg; pre-hypertensive, 120–139 and/ or 80–89 mmHg; hypertensive, ≥140 or ≥90 mmHg.

**Table 5:** Odds ratios with 95% CIs for cut-off points of carotid artery characteristics among groups according to blood pressure category after multivariate adjustment.

Variables	Model 1 OR (95% CI)			Model 2 OR (95% CI)			Model 3 OR (95% CI)		
	Normotensive	Pre-Hypertensive	Hypertensive	Normotensive	Pre-Hypertensive	Hypertensive	Normotensive	Pre-Hypertensive	Hypertensive
IMT_max_ (cut-off: ≥1.00 mm)	1.0 (ref.)	5.40 ** (1.786–16.536)	14.72 *** (4.952–43.730)	1.0 (ref.)	4.20 *** (1.292–13.652)	10.65 *** (3.426–33.118)	1.0 (ref.)	2.30 (0.688–7.714)	5.90 ** (1.833–18.968)
LD_max_ (cut-off: ≥0.68 cm)	1.0 (ref.)	3.32 *** (1.897–5.807)	3.73 *** (2.043–6.798)	1.0 (ref.)	2.70 ** (1.494–4.885)	3.04 ** (1.606–5.770)	1.0 (ref.)	2.10 * (1.133–3.888)	2.38 * (1.226–4.614)
LD_min_ (cut-off: ≥0.63 cm)	1.0 (ref.)	6.01 *** (2.527–7.756)	4.43 *** (3.304–10.938)	1.0 (ref.)	3.52 *** (1.953–6.353)	4.69 *** (2.486–8.828)	1.0 (ref.)	2.55 *** (1.7902–6.632)	3.45 ** (1.380–4.713)
PFV (cut-off: ≤57.5 cm/s)	1.0 (ref.)	3.14 *** (2.262–5.669)	3.18 *** (2.312–6.493)	1.0 (ref.)	2.41 ** (1.332–4.373)	2.64 ** (1.519–4.597)	1.0 (ref.)	1.68 (0.931–3.025)	1.62 (0.871–3.029)
EFV (cut-off: ≤19.5 cm/s)	1.0 (ref.)	3.78 *** (2.221–6.434)	4.53 *** (2.558–8.036)	1.0 (ref.)	3.06 *** (1.751–5.331)	3.36 *** (1.845–6.106)	1.0 (ref.)	1.96 * (1.086–3.545)	2.29 * (1.220–4.286)
PFV/LD_min_ (cut-off: ≤94.0 s^−1^)	1.0 (ref.)	4.38 *** (2.527–7.605)	5.16 *** (2.863–9.316)	1.0 (ref.)	3.56 *** (2.004–6.306)	3.90 *** (2.116–7.178)	1.0 (ref.)	2.20 * (1.194–4.035)	2.55 ** (1.341–4.832)
EFV/LD_max_ (cut-off: ≤30.0 s^−1^)	1.0 (ref.)	4.25 *** (2.377–7.597)	7.06 *** (3.848–12.967)	1.0 (ref.)	3.29 *** (1.793–6.034)	5.32 *** (2.826–10.015)	1.0 (ref.)	2.04 * (1.077–3.870)	3.58 *** (1.852–6.921)
CAC, % 10 mmHg (cut-off: ≤0.75 % 10 mmHg)	1.0 (ref.)	2.27 ** (1.373–3.738)	3.22 *** (1.870–5.534)	1.0 (ref.)	2.02 ** (1.192–3.404)	2.93 *** (1.657–5.179)	1.0 (ref.)	1.50 (0.865–2.590)	2.17 * (1.204–3.915)
β-stiffness index, AU (cut-off: ≥12.9 AU)	1.0 (ref.)	1.94 ** (1.183–3.186)	2.68 *** (1.563–4.588)	1.0 (ref.)	1.84 * (1.089–3.094)	2.65 ** (1.495–4.670)	1.0 (ref.)	1.41 (0.815–2.422)	2.02 * (1.120–3.628)

OR, odds ratio; CI, confidence interval; IMT, intima–media thickness; LD, luminal diameter; PFV, peak-systolic flow velocity; EFV, end-diastolic flow velocity; CAC, carotid artery compliance. Three models were used for the multinomial logistic regression: Model 1, adjusted for cardiovascular disease, stroke, lung disease, musculoskeletal disorders, diabetes, hyperlipidemia, current alcohol drinking, current smoker, and sex; Model 2, adjusted for Model 1, plus, body mass index, body fat percent, lean body mass, waist to hip ratio, and physical activity; Model 3, adjusted for Models 1 and 2, plus, and age. * *p* < 0.05, ** *p* < 0.1, *** *p* < 0.001.
